# Optogenetic stimulation of Drosophila heart rate at different temperatures and Ca^2+^ concentrations

**DOI:** 10.14814/phy2.12695

**Published:** 2016-02-03

**Authors:** Yue C. Zhu, Henry Uradu, Zana R. Majeed, Robin L. Cooper

**Affiliations:** ^1^ Department of Biology and Center for Muscle BiologyUniversity of KentuckyLexingtonKentucky; ^2^ Department of BiologyCollege of ScienceUniversity of SalahaddinErbilIraq

**Keywords:** Cardiac, Drosophila, optogenetics

## Abstract

Optogenetics is a revolutionary technique that enables noninvasive activation of electrically excitable cells. In mammals, heart rate has traditionally been modulated with pharmacological agents or direct stimulation of cardiac tissue with electrodes. However, implanted wires have been known to cause physical damage and damage from electrical currents. Here, we describe a proof of concept to optically drive cardiac function in a model organism, *Drosophila melanogaster*. We expressed the light sensitive channelrhodopsin protein ChR2.XXL in larval *Drosophila* hearts and examined light‐induced activation of cardiac tissue. After demonstrating optical stimulation of larval heart rate, the approach was tested at low temperature and low calcium levels to simulate mammalian heart transplant conditions. Optical activation of ChR2.XXL substantially increased heart rate in all conditions. We have developed a system that can be instrumental in characterizing the physiology of optogenetically controlled cardiac function with an intact heart.

## Introduction

Pharmacology is the front‐line treatment for intervening in cardiac pathologies that impair heart rate. In addition or in combination with drug applications, direct electrical control of the heart through pacemakers is also widely practiced. Pacing of human hearts with electrical stimulating pacemakers or implantable cardioverter defibrillators – which are placed on or nearby cardiac tissue – have saved and prolonged many lives. However, in some cases, these implanted devices can cause irritation and damage to cardiac tissue over time, requiring additional surgeries (Kaye et al. 1975; Stefanidis et al. 2009; Wolk et al. 2013). Novel approaches to control cardiac function are now possible with gene therapy or a combination of gene therapy and regulated gene expression (Lee et al. 1999), although there are various results that warrant caution regarding the safety and efficacy of gene therapy (Giacca and Baker 2011; Jessup et al. 2011). Clearly, more research is needed to enhance the tools and approaches to gene therapy in cardiac tissue to make effective, customized treatments a reality.

One of the gene therapy interventions being considered in cardiac medicine is optogenetics. Light‐controlled pacemaker cells could be an effective alternative to implanted electrode pacemakers. Basic research in cardiac optogenetics is needed to gather information about the physiology of optically stimulated heart tissue. Fruit flies are an attractive model system to efficiently gather molecular data on the use of optogenetics on the heart. *Drosophila melanogaster* has been used extensively to investigate cardiac function in response to chemical modulators (Titlow et al. 2013; Majeed et al. 2014; Malloy et al. 2016), environmental changes (stress, temperature), (de Castro et al. 2014) as well as developmental abnormalities and cellular regulation of Ca^2+^ dynamics (Johnson et al. 1998; Desai‐Shah et al. 2010; Becnel et al. 2013). Although the anatomy of the larval heart tube in *Drosophila* is quite different from the chambered heart of vertebrates, the key molecular mechanisms in heart development and function are shared to a large degree between the two (Bodmer and Venkatesh 1998). In addition, the physiological parameters of cardiac output, rate, and duration of systole and diastole between *Drosophila* and vertebrates are quite comparable (Choma et al. 2011). The larval *Drosophila* heart is myogenic and pacing is not regulated by direct neural connections. The vast collection of genetically engineered lines for controlling cellular activity and manipulating gene expression make *Drosophila* a powerful model system (Jones 2009; Yoshihara and Ito 2012). The use of optogenetics in *Drosophila* has already been optimized for altering specific behaviors by driving channelrhodopsins in identifiable sets of neurons (Owald et al. 2015; Titlow et al. 2015).

Here, we demonstrate that heart rate can be elevated in the *Drosophila* larva heart using the light‐sensitive channel rhodopsineChR2.XXL, a hypersensitive variant of ChR2 (Alex et al. 2015). Our approach allowed us to examine the effects of ion exchange and bath temperature on the efficacy of optical stimulation. The motivation for testing different conditions was to assess the plausibility of using optogenetics in conditions suitable for human heart transplants. The immersion saline used to maintain tissue viability for human transplants, called “Celsior” (Michel et al. 2014), has low Ca^2+^ concentration and is used at low temperatures, conditions which could impair the channelrhodopsin cation channel. Thus, to explore the potential for using light as a pacemaker in mammalian heart transplant conditions, we investigated optogenetic stimulation of the heart over a range of bath temperatures and Ca^2+^concentrations.

## Methods and Methods

### Drosophila lines

The filial 1 (F1) generations were obtained by crossing females of the recently created ChR2 line (which is very sensitive to light) called y^1^ w^1118^; PBac{UAS‐ChR2.XXL}VK00018 (BDSC stock # 58374) (Dawydow et al. 2014) with male 24B‐Gal4 (III) (BDSC stock # 1767). These background strains were also examined for the effect of light on the heart.

### Preparation of fly food supplemented with ATR

All trans retinal (ATR; Sigma‐Aldrich, St. Loius, MO, USA) was diluted in 50 ml of standard fly food to a final concentration of 200 *μ*M and protected from light with aluminum foil. For control experiments, larvae were cultured in food that only contained the solvent (100 *μ*l of absolute ethanol in 50 ml of fly food).

### Heart rate measurement and experimental conditions

Larvae were dissected in a modified hemolymph‐like 3 (HL3) solution (Stewart et al. 1994) containing: (in mmol/L) 70 NaCl, 5 KCl, 20 MgCl_2_, 10 NaHCO_3_, 1 (or 0.5 mmol/L) CaCl_2_, 5 trehalose, 115 sucrose, 25 N,N‐Bis‐(2‐hydroxyethyl)‐2‐aminoethane sulfonic acid (BES). Heart rate in *Drosophila* larvae is very sensitive to pH changes, thus, the saline requires substantial buffering to reduce drift in pH. The following modifications to the HL3 saline were made: a decrease in pH from 7.2 to 7.1, and increase in BES buffer from 5.0 mmol/L to 25.0 mmol/L to maintain a stable pH (de Castro et al. 2014).

Larvae were either raised at room temperature 21°C or placed at 10°C as second instar for 10 days prior to experimentation (herein, referred to as cold conditioned). When the larvae reached a third instar stage, they were used. Cold‐conditioned larvae were dissected in a 10°C cold room and tested in 10°C saline. Room temperature larvae were dissected at 21°C and exposed to saline at the same temperature. A detailed description of the dissection protocol is shown in video format (Cooper et al. 2009). In brief, larvae were slit along the mid‐ventral longitudinal axis and pinned flat. The internal organs and GI tract were removed and care was given not to damage either the trachea or heart. The brain was left intact as the anterior end of the heart tube is attached to the connective tissue around the brain. With the heart exposed, fresh saline was applied and the contraction rate was counted at the most caudal end. Heart rate (HR) was counted after allowing the preparation to remain still for 1 min and counted in the subsequent minute under dim white light. For optogenetic experiments, a continuous beam of blue light (470 nm wavelength) from a high intensity LED was focused on the specimen through a 10× ocular objective while the HR was counted (Titlow et al. 2014).

### Statistical analysis

All data are expressed as raw values or mean ± SEM. A paired *t*‐test (before and after) or a rank sum pairwise test was used to analyze changes in HR after changing bath conditions or stimulating with blue light. On the bar charts of the raw data for HR, a star with a T (*T) indicates a significant difference *P* < 0.05 for the *T*‐test. Horizontal lines above the bar charts indicate significant differences (*P* < 0.05) among the groups by a Dunn's Test of rank differences. Since the groups are not normally distributed (a number of zeros in some groups) and having different sample sizes a Dunn's Test of rank differences appears to be a feasible approach.

## Results

### Extracellular calcium levels regulate heart rate in cold‐conditioned larvae

**Figure 1:**
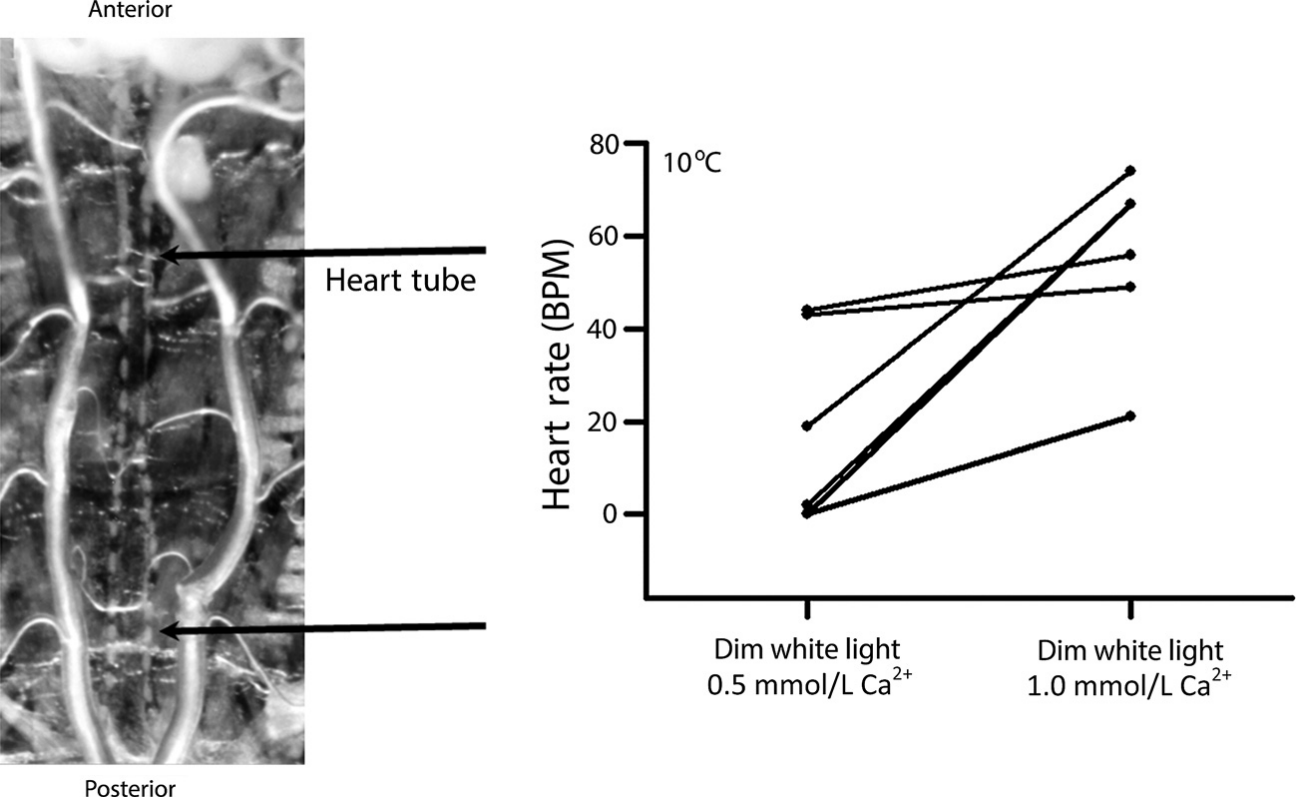
Heart rate (HR) is regulated by extracellular calcium concentration in cold‐conditioned larvae (10°C). (A) The larva heart tube is located between the two trachea in a dissected preparation. The posterior region is the true heart where the rates are measured. (B) Changing the buffer from 0.5 to 1.0 mmol/L Ca^2+^ increased HR in all preparations (*P* < 0.05, nonparametric analysis). These are the control background strain UAS‐ChR2.XXL fed ATR.

Heart transplants from mammalian donors are kept at low temperature in a low calcium buffer to reduce metabolic damage and increase viability. To develop an assay system that simulates those conditions we cold conditioned *Drosophila* larvae to 10°C and analyzed heart function with the tissue bathed in 10°C physiological saline at different calcium concentrations (Fig. 1). Larval hemolymph has a Ca^2+^ concentration between 1.0 and 1.5 mmol/L (Stewart et al. 1994). We investigated heart function at lower Ca^2+^ concentrations to compare with the mammalian Celsior buffer, which has a Ca^2+^ of 0.25 mmol/L. At 10°C, some hearts did not beat in saline with 0.5 mmol/L [Ca^2+^], but began beating after calcium levels were increased to 1.0 mmol/L. Increasing [Ca^2+^] from 0.5 to 1.0 mmol/L caused HR to increase in all six experiments tested (Fig. 1). Therefore, these calcium conditions were used for subsequent experiments.

### Optogenetic stimulation increases larval heart rate

**Figure 2:**
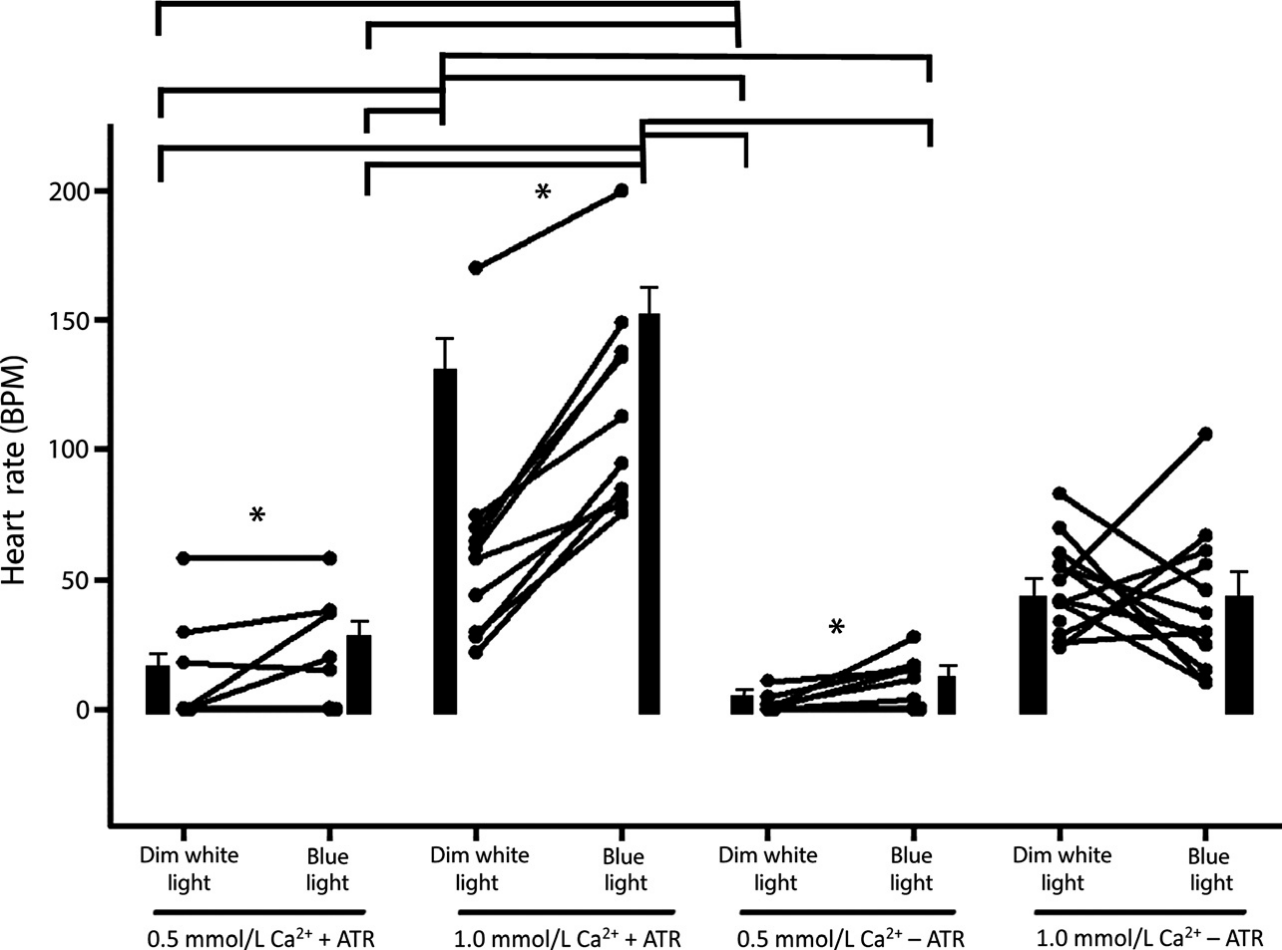
Optogenetic stimulation of larval heart rate (HR) at room temperature (21°C). HRs measured in individual larvae in 0.5 mmol/L and 1.0 mmol/L Ca^2+^ with or without food supplemented with ATR. The only condition which did not show a significant increase in HR is 1.0 mmol/L Ca^2+^ without ATR (−ATR) (star is significant, nonparametric *P* < 0.05). The mean HR (±SEM) for each condition is also shown (solid bars). Horizontal lines above the bar charts indicate significant differences (*P* < 0.05) among the groups by a Dunn's Test of rank differences.

To determine if optogenetics could be used to stimulate heart contractions, we expressed ChR2.XXL (24B‐Gal4 > Chr2.XXL) in heart tissue and counted HRs before and during continuous exposure to blue light. In each of the 10 preparations tested, continuous optical stimulation caused the HR to increase (Fig. 2; 1.0 mmol/L Ca^2+^, +ATR). The effect of optical stimulation was substantially reduced in low calcium saline (Fig. 2; 0.5 mmol/L, +ATR), with only 3/9 preparations showing an increase in HR. Hearts that were not beating in 0.5 mmol/L Ca^2+^ began to beat rapidly during exposure to blue light.

As a negative control, HRs were recorded in larvae that were not pretreated with the ATR cofactor. During optical stimulation in these larvae, HR increased for 8/11 and 6/11 specimens in normal and low calcium conditions, respectively (Fig. 2; 1.0 mmol/L Ca^2+^ −ATR and 0.5 mmol/L Ca^2+^ −ATR). This result suggests that there could be a low level of ChR2.XXL activity in the heart even without the co‐factor. As an additional negative control, we tested genetically related strains that were fed ATR, but did not express ChR2.XXL (24B‐Gal4/+ and UAS‐ChR2.XXL/+). Optical stimulation had negligible effects on HR in these strains, that is, during stimulation the average HR for these lines changed from 159 ± 8 BPM to 155 ± 5 BPM (24B‐Gal4/+) and from 189 ± 12B PM to 194 ± 15 BPM (UAS‐ChR2.XXL/+), respectively. The absence of a response in these lines combined with the robust response to optical stimulation at normal calcium levels when ChR2.XXL was expressed in the heart tissue shows that optogenetic stimulation reliably elevates HR in the dissected larva preparation.

### Optogenetic heart stimulation is effective in cold‐conditioned larvae

**Figure 3:**
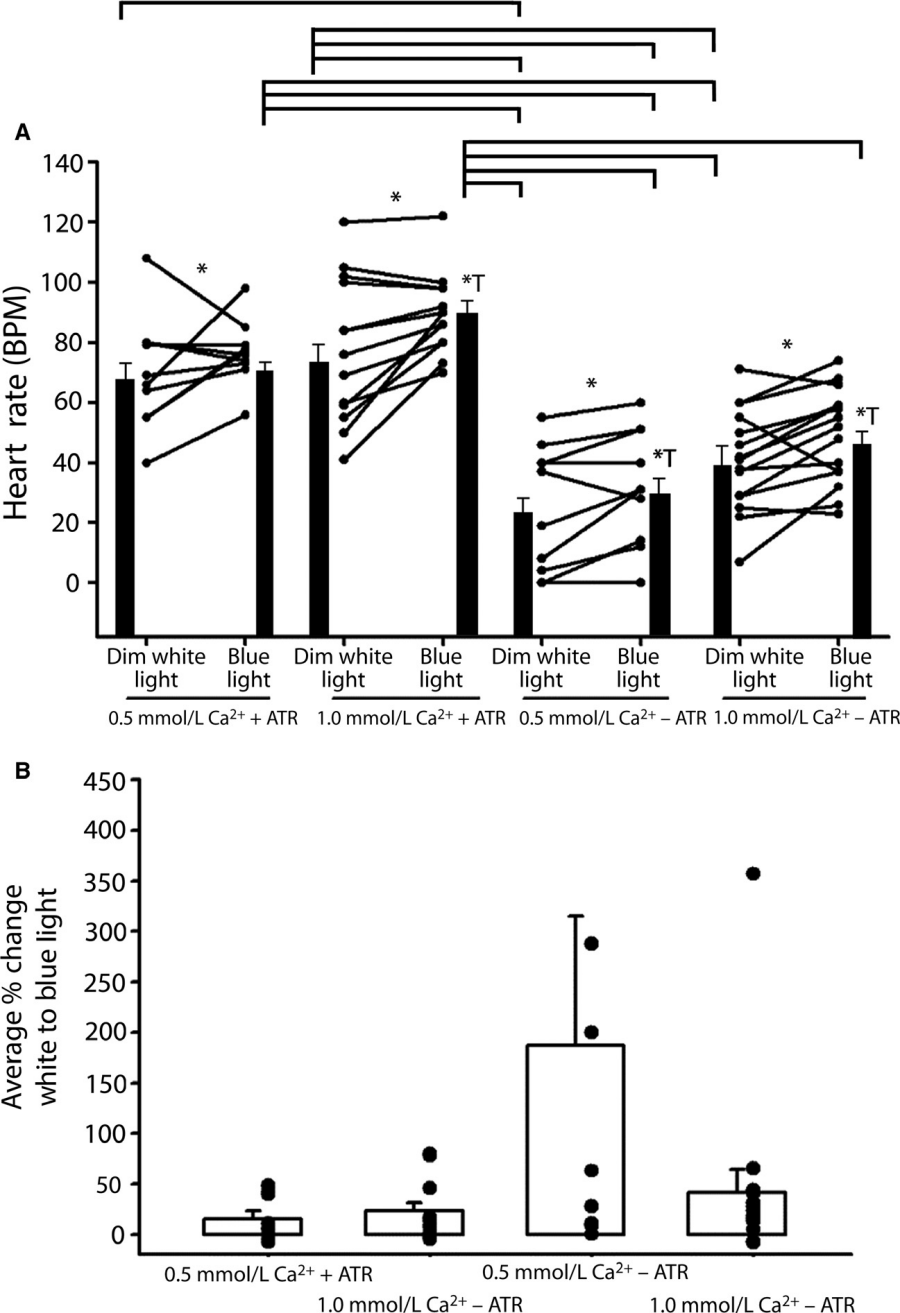
Optogenetic stimulation of heart rate (HR) in cold‐conditioned larvae (10°C). (A) HRs measured in individual larvae in 0.5 mmol/L and 1.0 mmol/L Ca^2+^ with or without food supplemented with ATR. All conditions show a significant increase in HR (star is significant, nonparametric *P* < 0.05); stars with a T are significant with measures as a before and after effect performed with a *T*‐test. All are significant except 0.5 mmol/L Ca^2+^ with ATR). The mean HR (±SEM) for each condition is also shown (solid bars). Horizontal lines above the bar charts indicate significant differences (*P* < 0.05) among the groups by a Dunn's Test of rank differences. (B) The percent change before and after focused blue light for each condition in (A) revealed a greater change for preparations not fed ATR (−ATR).

To further test the possibility of using this assay in conditions that simulate mammalian heart transplants, we characterized the efficacy of optogenetic heart stimulation in cold‐conditioned larvae. Not unexpectedly, the effect of optical stimulation on larval HR was slightly reduced at 10°C, but HR increased in 10/13 preparations tested (Fig. 3A; 1.0 mmol/L Ca^2+^, +ATR). As in the room temperature experiments, optical stimulation of HR was calcium dependent. HR increased in fewer preparations and exhibited a smaller percent change at 0.5 mmol/L Ca^2+^ than at 1 mmol/L Ca^2+^ (Fig. 3B). Also, similar to room temperature experiments, we observed an optical response in the absence of ATR, again suggesting that ChR2.XXL could be active without the co‐factor. Nonetheless, these results show that ChR2.XXL is functional at low temperatures in the heart and that they can be activated under low calcium conditions.

## Discussion

The possibility of optically stimulating cardiac tissue in lieu of electrical stimulation provides alternative approaches for regulation of cardiac function within an animal as well as regulation of isolated tissue. In this study, we illustrate the potential for optogenetics to be used in conditions suitable for human heart transplants and/or mammalian cardiac tissue cell culture. We demonstrated the use of optogenetics in various conditions to gain a better understanding of the chemical and physical limits of optically driving the cardiac tissue to pace. Activity of the Gal4 transcriptional activator decreases when flies are raised at low temperature (Duffy 2002). This study shows that even at low temperature, the channelrhodopsin‐XXL is robustly activated in the cardiac tissue. Ultimately, these experiments show that the ChR2.XXL channelrhodopsin is functional down to 10°C and 0.5 mmol/L Ca^2+^.

Exposed hearts in filleted larval *Drosophila* preparations with defined saline were used, so, we could control the ionic media and rapidly shift the temperature without additional variables of hormones or other unknown hemolymph‐borne factors within intact larvae. In the dissected cardiac preparations, the heart tube is flooded with saline magnitudes of volume greater than the volume of hemolymph in the intact larva and the saline is exchanged after the dissection. So, any light‐induced stress which may induce the release of hormones within the larvae would have negligible effects on the heart. The controlled concentration of calcium in the saline, temperature, and the ATR food supplement all showed significant effects depending on the experimental conditions.

An important consideration in optogenetic stimulation of the heart and other tissues is photosensitivity of the channelrhodopsins. Larvae expressing the ChR2.XXL were so sensitive to light that HR was elevated during exposure to a dim fiber optic white light source. After dark conditioning and then immediately counting HR under dim white light, the HR increases rapidly and reaches a steady state. That is why, larvae pretreated with ATR exhibited higher baseline HRs than untreated controls for both calcium conditions and at both temperatures. On top of this background sensitivity to unfiltered light, we still observed significant effects for 470 nm light on HR. We are currently developing a high‐throughput approach to electrically monitor HR in the absence of light to eliminate this problem and to determine the wavelength specificity for channelrhodopsins expressed in larval heart tissue. The hypersensitive ChR2.XXL channel may prove to be more efficacious in translational science to intact mammals than previous versions of ChR2 due to its increased photosensitivity. In activating mammalian cardiac tissue in vivo, light would likely need to penetrate fluid bathing the heart either within the pericardial sac or within the cardiac chambers. The light would be dampened in the fluid, thus higher photosensitivity may be necessary.

Direct optogenetic pacing of the *Drosophila* heart in larvae, pupa, and adult stages was recently reported by Alex et al. (2015). This study complements their noninvasive approach by enabling direct access to the tissue for pharmacology and ion exchange. This adds another excellent tool to the fly genetic toolbox, which can be used to study various physiological aspects of heart rate at low temperatures.

## Conflict of Interest

None declared.
